# Genome-Wide Bovine H3K27me3 Modifications and the Regulatory Effects on Genes Expressions in Peripheral Blood Lymphocytes

**DOI:** 10.1371/journal.pone.0039094

**Published:** 2012-06-28

**Authors:** Yanghua He, Ying Yu, Yuan Zhang, Jiuzhou Song, Apratim Mitra, Yi Zhang, Yachun Wang, Dongxiao Sun, Shengli Zhang

**Affiliations:** 1 Key Laboratory of Agricultural Animal Genetics and Breeding, National Engineering Laboratory for Animal Breeding, College of Animal Science and Technology, China Agricultural University, Beijing, P.R. China; 2 Department of Animal and Avian Sciences, University of Maryland, College Park, Maryland, United States of America; Queen’s University Belfast, United Kingdom

## Abstract

**Background:**

Gene expression of lymphocytes was found to be influenced by histone methylation in mammals and trimethylation of lysine 27 on histone H3 (H3K27me3) normally represses genes expressions. Peripheral blood lymphocytes are the main source of somatic cells in the milk of dairy cows that vary frequently in response to the infection or injury of mammary gland and number of parities.

**Methods:**

The genome-wide status of H3K27me3 modifications on blood lymphocytes in lactating Holsteins was performed via ChIP-Seq approach. Combined with digital gene expression (DGE) technique, the regulation effects of H3K27me3 on genes expressions were analyzed.

**Results:**

The ChIP-seq results showed that the peaks of H3K27me3 in cows lymphocytes were mainly enriched in the regions of up20K (∼50%), down20K (∼30%) and intron (∼28%) of the genes. Only ∼3% peaks were enriched in exon regions. Moreover, the highest H3K27me3 modification levels were mainly around the 2 Kb upstream of transcriptional start sites (TSS) of the genes. Using conjoint analysis with DGE data, we found that H3K27me3 marks tended to repress target genes expressions throughout whole gene regions especially acting on the promoter region. A total of 53 differential expressed genes were detected in third parity cows compared to first parity, and the 25 down-regulated genes (*PSEN2 etc.*) were negatively correlated with H3K27me3 levels on up2Kb to up1Kb of the genes, while the up-regulated genes were not showed in this relationship.

**Conclusions:**

The first blueprint of bovine H3K27me3 marks that mediates gene silencing was generated. H3K27me3 plays its repressed role mainly in the regulatory region in bovine lymphocytes. The up2Kb to up1Kb region of the down-regulated genes in third parity cows could be potential target of H3K27me3 regulation. Further studies are warranted to understand the regulation mechanisms of H3K27me3 on somatic cell count increases and milk losses in latter parities of cows.

## Introduction

Dairy cows provide a mass of milk and protein for the human beings world-wide. High quality milk is required for human health. For dairy cattle, ten or more lactations are possible and the highest milk production of dairy cows should be at the fifth parity from physiological perspective. However, the average herd life of Holsteins is under three lactations at present [1]. Studies reported that over 90% of all cows are culled due to reasons of infertility, mastitis, lameness and lower milk production [2], [3], [4]. Although somatic cells are normal constituent of milk, high somatic cell counts (SCC) in the milk have been shown to be significantly related with milk quality, milk losses and mastitis risk [5], [6].

Peripheral blood lymphocytes are the main source of somatic cells in the milk of dairy cows in response to the infection or injury of the mammary gland [7]. Lymphocytes include three major types of cells, natural killer cells, T cells and B cells. Natural killer cells are a part of the innate immune system, whereas, T cells and B cells are the major components of the adaptive immune response [8]. Proper lymphocyte development and function are the result of multiple cell lineage choices and cellular transitions with the hematopoietic system. Numerous studies are working on understanding the contribution of transcriptional and epigenetic regulation during lymphopoiesis at both physiological and pathological levels in recent years [9], [10], [11], [12], [13].

The complex relationships between transcription level and the epigenetic machinery of lymphocytes identity and development are partially revealed [12], [14], [15]. Posttranslational histone modifications are the major part of epigenetics, which include acetylation, methylation, phosphorylation and ubiquitination [16]. Acetylation is the most frequently studied histone modification and is commonly localized to active chromatin. However, histone methylation plays a dual role as it is important in both activation and repression, depending on where the methyl group is modified. Trimethylation of lysine four on histone H3 (H3K4me3) is associated with transcriptional activity [17]. By contrast, trimethylaiton of lysine 27 on histone H3 (H3K27me3) is related with inactivation of genes expressions or located in heterochromatin [18], [19]. Studies reported that H3K27me3 are relatively stable through lymphocyte divisions and for preserving cell identity, and are therefore considered as potential marks for transmitting the epigenetic information [20], [21].

In bovine, the remodeling of H3K27me3 was investigated during development of *in vitro* fertilization (IVF) and somatic cell nuclear transfer (SCNT) bovine embryos [22], [23]. The global dynamics of H3K27me3 during bovine oocyte maturation and preimplantation development were detected by semiquantitative immunofluorescence [24]. However, genome-wide H3K27me3 modifications and regulatory function are still unclear in bovine lymphocytes. The objectives of the study were to map the genome-wide H3K27me3 landscape of bovine via high-throughput chromatin immunoprecipitations coupled with sequencing (ChIP-Seq) approach, and to declare the regulatory effects of H3K27me3 modification on genes expressions in peripheral blood lymphocytes by means of digital gene expression (DGE) techniques. The potential functions of H3K27me3 modification on the parity-associated differentially expressed genes were also analyzed and discussed.

## Results

### Genome-wide Maps of H3K27me3 Modifications in Cows Lymphocytes

**Figure 1:**
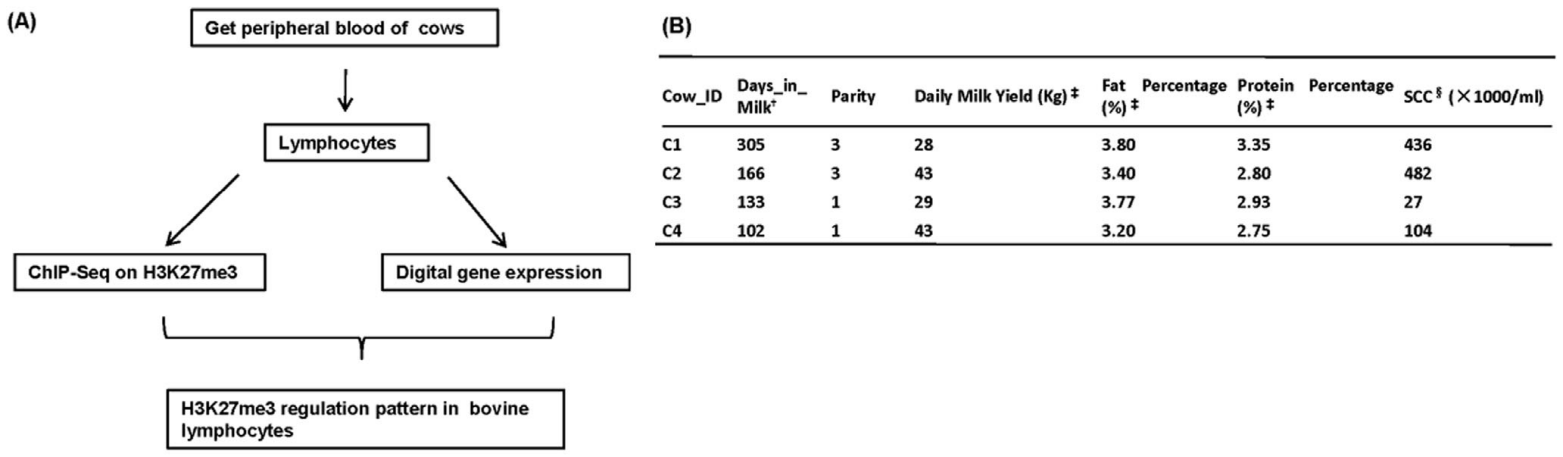
Overview of the experimental design and samples characterization. (A) Peripheral blood was collected from cow caudal vein, and primary lymphocytes were isolated. The genome-wide distribution of H3K27me3 was determined with ChIP-seq, and all genes expressions in lymphocytes were performed with Digital Gene Expression technique. (B) Performance testing information about the four cows. ^†^Days_in_Milk is the days from calving to sampling. ^‡^Daily milk yield, fat percentage and protein percentage were the average values of annual records. ^§^Somatic cell counts.

To reveal epigenetic features of lymphocytes in bovine peripheral blood, we generated global maps of H3K27me3 modifications via the ChIP-seq approach. A total of 9.7 million short reads from each sample were aligned to the bovine reference genome (Btau4.0). We utilized the MACS model-based algorithm for identification of significant clustering of windows with enriched ChIP signals, which were called “peaks” of H3K27me3 in the study. The scaling analysis indicated that the sequence reads were sufficient to identify most regions associated with the H3K27me3 modifications in the bovine genome [25] (see Figure S1 for details). To understand the functional consequences of H3K27me3 modification, gene expression in bovine lymphocytes were analyzed by the DGE assay. Therefore, we made a combined analysis for ChIP-seq and DGE to gain insights into the H3K27me3 regulation pattern in bovine lymphocytes (Figure 1).

#### Distribution of H3K27me3 reads

**Figure 2:**
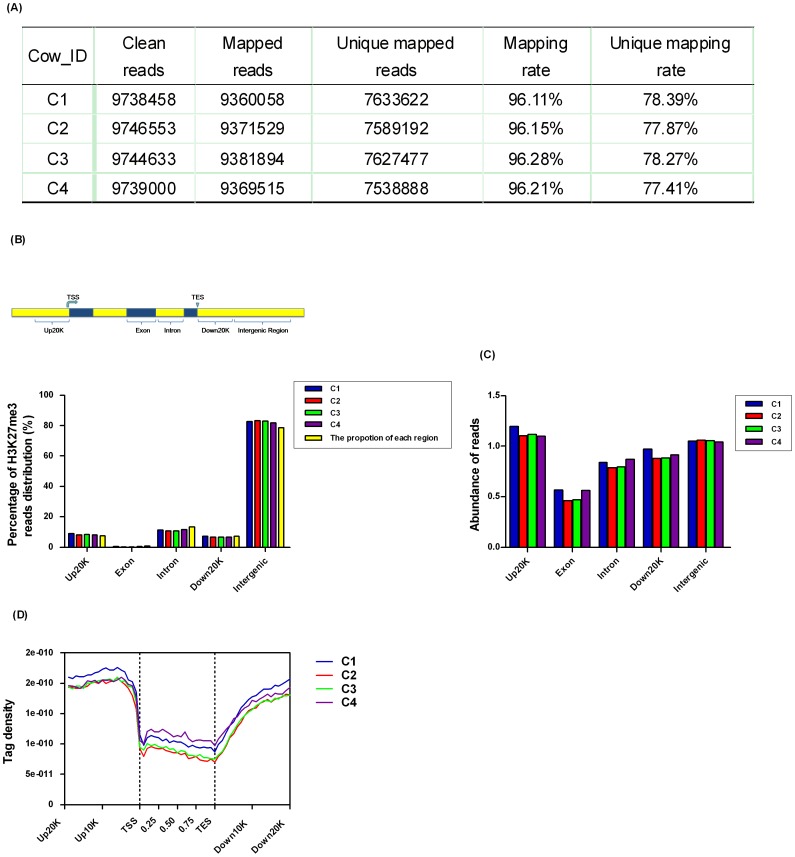
Distribution of bovine H3K27me3 reads. (A) The mapping result of H3K27me3 reads. Raw reads were generated by Solexa sequencing, and then clean reads were obtained after filtering dirty reads. All clean reads were mapped to the bovine reference genome and only uniquely matching reads were retained to use for subsequent analysis. Mapping rate is the ratio of mapped reads to clean reads and unique mapping rate is the ratio of unique mapped reads to clean reads. (B) Distribution of H3K27me3 reads among different genomic regions. The bovine genome was divided into five kinds of regions: 20 kb upstream of transcription start site (TSS), exon, intron, 20 kb downstream of transcription end site (TES) and intergenic regions. The histogram described the percentage of unique mapped reads among five genomic regions and the proportion of each region of the total genome. (C) Abundance of H3K27me3 reads among different genomic regions. The percentages of reads distribution were normalized to the abundance values. (D) Coverage depth of H3K27me3 reads among genic regions. For each gene, the tag numbers detected in every 5% of the gene-body region and every 1 kb outside of the gene-body region were summed to obtain methylation levels. These numbers were then normalized by the total number of base pairs in each region [20].

To obtain an overall picture of H3K27me3 distribution, all reads of ChIP-seq were aligned to the bovine genome and only uniquely matching reads were retained after filtering dirty reads. Mapping rate was 96% and unique mapping rate was 77%, which laid down a basis for further analysis (Figure 2A). To study H3K27me3 distribution in genome region, we divided the bovine genome into five kinds of regions–up20K (20 kb upstream of transcription start site (TSS)), exon, intron, down20K (20 kb downstream of transcription end site (TES)) and intergenic regions–on the basis of annotation of “known genes” from BioMart Query System of ENSEMBL Btau4.0 database developed jointly by the OICR and the EBI. The proportion of each region of the total genome was indicated (Figure 2B). As shown in Figure 2B, more reads were identified in intergenic regions (∼83%) and few reads in exon regions (∼0.35%). However, their proportions of the total genome were different with intergenic of 78.7% and exon regions of 0.71%. Reads located in up20K and down20K were 8.4% and 6.7%, but their proportions of the total genome were the same (7.4%). For intron region, approximately reads of 11% were distributed and this region accounted for the entire genome of 13%. In view of this point, abundance of reads located in these regions was drawn in Figure 2C. It showed that the abundance of up20K was highest and that of exon region was lowest. To visualize the distribution trends of H3K27me3 in the genic regions, a composite profile of H3K27me3 for all known genes was generated, spanning their gene bodies and extending 20 kb upstream and 20 kb downstream (Figure 2D). It is notable that H3K27me3 signals levels were high on upstream20Kb of TSS. Moreover, it appeared that H3K27me3 distribution decreased dramatically within gene bodies, in accordance with the observation that less of the H3K27me3 reads were located in exon regions (Figure 2B). These results were consistent with previous observations that H3K27me3 associates extensively with proximal promoters in human T cells, as well as human and murine embryonic stem cells [25], [26], [27].

#### Distribution of H3K27me3 peaks

**Figure 3:**
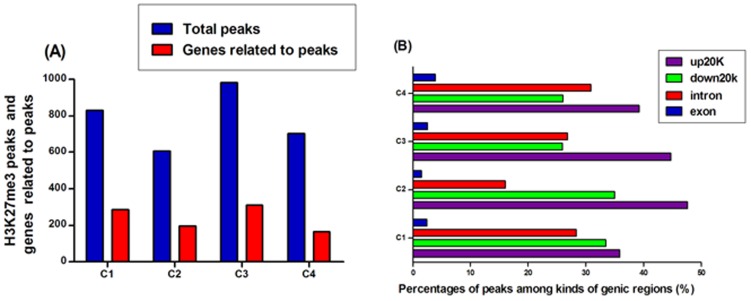
Distribution of bovine H3K27me3 peaks. (A) H3K27me3 peaks and genes related to peaks. (B) Percentages of genes related to H3K27me3 peaks. The bovine genic region was divided into four kinds of regions: 20 kb upstream of TSS, exon, intron, and 20 kb downstream of TES. The bar chart described the percentage of genes associated with H3K27me3 peaks among kinds of genic regions.

As the final ChIP DNA fragments contain immunoprecipitated target DNA only at minor fraction (reportedly <1%) within a large proportion of background input DNA [28], [29], many researchers seek to identify locations of ChIP target DNA in which sequence reads cluster into many enrichment regions, wherein overlapping reads appear as peaks. In the present study, MACS1.4.0 software was used to identify enriched ChIP regions of H3K27me3. In total, 831, 607, 982 and 703 peaks were found, while the known genes related to peaks were 285, 195, 311 and 165 in the four cows, respectively (Figure 3A). To study the pattern of epigenetic modifications in different regions of genes, we also calculated distribution percentages of peaks identified in four kinds of genic regions. The result showed that most peaks enriched in gene regions of up20K (∼50%), down20K (∼30%) and intron (∼28%), whereas only 3% peaks were enriched in exon regions (Figure 3B), suggesting that H3K27me3 modification in bovine lymphocytes may play a major role in regulatory region of genes. It is worth mentioning that a higher percentage of enrichment in up20K of genes was marked with H3K27me3 modification compared to in down20K region, consistent with the observation that more H3K27me3 enrichments were located 5 Kb upstream of TSS than in 5 Kb downstream of TES in murine T cell subset [25]. In summary, our data indicated that high H3K27me3 modification levels of bovine lymphocytes were mainly in regulatory regions of genes.

### Correlation of H3K27me3 Modifications with Gene Expression

To reveal the functional consequences of H3K27me3 on target genes, we generated expression profiles for the four individuals’ lymphocytes using DGE tag profiling technology, which uses Illumina high-throughput sequencing to generate a large number of tags of length 21 bp. For the four individuals, 3.66×10^6^, 3.52×10^6^, 3.55×10^6^ and 3.64×10^6^ raw tags were generated, and the numbers of unambiguous tag-mapped genes were 6862, 6894, 6764 and 6923, respectively.

#### Correlation of H3K27me3 modifications near TSS with gene expression

**Figure 4:**
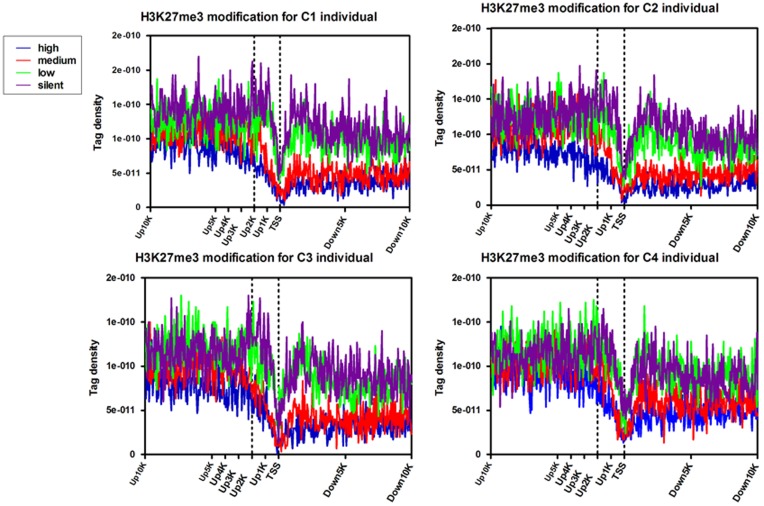
Modifications of H3K27me3 near transcription start sites. Profiles of the H3K27me3 indicated across the TSS for highly active (high), two kinds of intermediately active (medium and low) and silent gene sets were shown. Each gene set included 700 genes according to their expression levels in primary lymphocytes of cow peripheral blood. Here, up and down 20 kb regions of 700 genes in each group were aligned relative to their TSSs (x axis). The y axis shows the detected tag density.

Gene promoters near TSS contain critical regulatory elements necessary for transcription. Above genome-wide analyses have revealed that H3K27me3 modification was enriched in the promoter regions of the target genes. To correlate this modification with gene transcription, the bovine genes whose expression levels were determined by the DGE assay were separated into four groups of around 700 genes in each group according to their expression levels. The H3K27me3 tag numbers in each region were calculated and normalized near the TSS for four sets genes corresponding to highly expressed, two types of intermediately expressed (medium and low) and silent genes (Figure 4, Table S1). As expected, H3K27me3 signals were correlated with gene repression (Figure 4), which is in agreement with previous studies in human T cell and murine embryonic stem cells [30], [31]. Obviously, H3K27me3 levels were higher at silent genes than at active genes. Intriguingly, H3K27me3 levels were elevated surrounding the TSSs and peaked at upstream 2 K of TSS for the silent genes sets (dotted line), though were not significant for the other three sets. In order to confirm it, 113 silent genes were selected randomly to analyze their H3K27me3 profiles in all four individuals. The result showed that H3K27me3 levels indeed rise surrounding of the upsteam2K of TSS (Figure S5D), suggesting that H3K27me3 repressed gene expression by acting on the surrounding of upstream2K. A significant dip in the signal was observed at the TSS (Figure 4), which may correlate with lower nucleosome density at this region [32]. Moreover, it is evident that the modification levels of up10K relative to TSS were higher than of downstream10K regions, which conform to the result of Figure 2C and suggest that H3K27me3 occurs mainly in gene promoter region.

#### Correlation of H3K27me3 modifications across transcribed regions with gene expression

**Figure 5:**
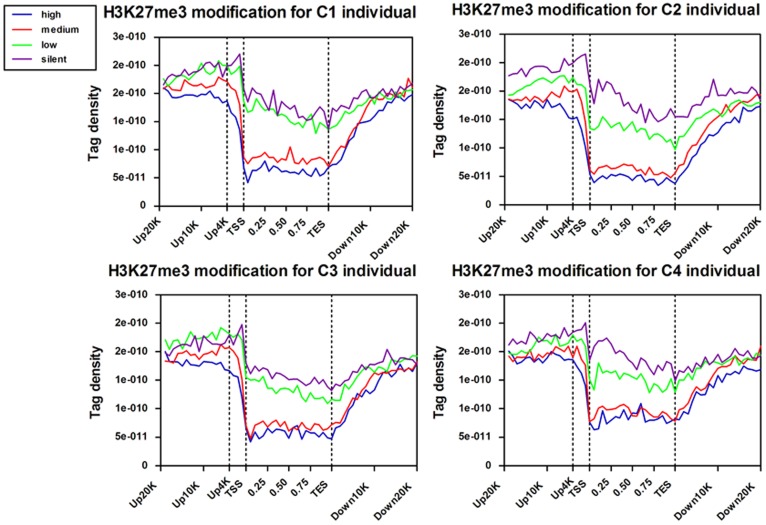
Modifications of H3K27me3 across the gene bodies. Profiles of H3K27me3 patterns of the four sets genes were shown across the gene bodies. The classification of four groups is the same as Figure 4 and density plots extend 20 kb of 5’ and 3’ of the gene bodies. For each gene, the tag numbers detected in every 5% of the gene-body region and every 1 kb outside of the gene-body region were summed to obtain methylation levels. These numbers were then normalized by the total number of base pairs in each region.

To reveal H3K27me3 features in transcribed regions of genes, we plotted their average modification levels in their transcribed regions and extending 20 kb upstream and 20 kb downstream for four sets genes. The combined analysis with H3K27me3 levels and genes expression profile showed that H3K27me3 marks tended to repress expressions of target genes across the whole gene regions (Figure 5, Table S2). Moreover, H3K27me3 were mainly enriched in upstream and downstream regions of the target genes, which is consistent with our above results and similar to H3K27me3 modifications in human T cell [20].

#### H3K27me3 modifications characteristics in cytokines and transcription factors genes

**Figure 6:**
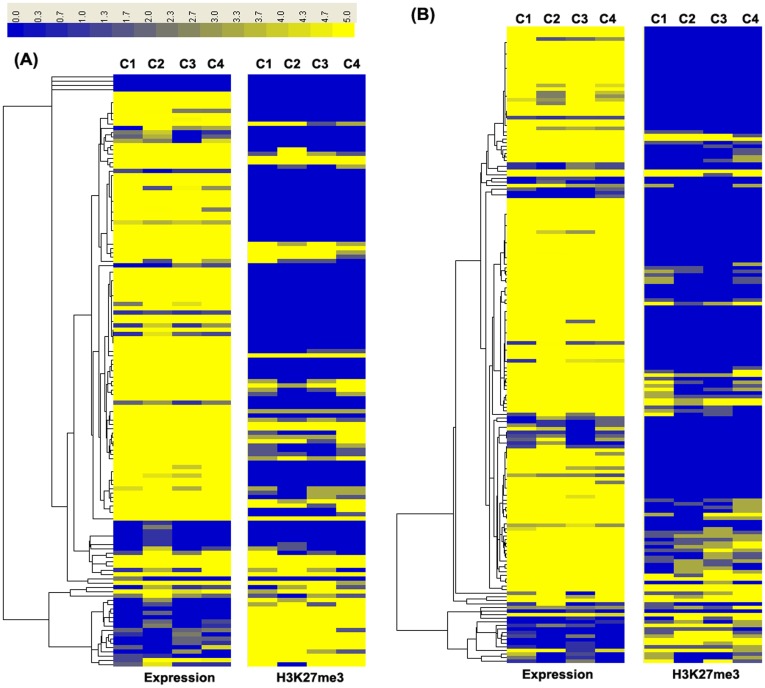
Hierarchical clustering of all cytokines and transcription factors genes. (A) Based on annotation for bovine DGE profiles, we identified 138 unique cytokine genes and their related genes. Normalized intensity values of genes (rows) and their H3K27me3 modifications were ordered using Centroid Spearman Rank Correlation and hierarchical clustering in Cluster3.0 software. The dendrogram showed the similarity (distance) of mRNA expression levels and H3K27me3 modifications of genes and was divided into sub-trees as distinguished from different colors. Arrays (columns) were grouped by four different individuals. Yellow and blue colors reflect the high and low intensities, respectively. (B) Based on annotation for bovine DGE profiles, 178 unique transcription factors and related genes were identified. The heatmap and the hierarchical cluster were generated as above described.

Different effectors of lymphocytes are characterized by lineage-specific expression of cytokine genes, and transcription factors play a role in this lineage commitment [33]. To further characterize the regulatory effects of H3K27me3 on the marked genes, we combined H3K27me3 maps with DGE data to identify groups of genes associated with cytokines and transcription factors. Two hierarchical clusters were generated: the first cluster contained all cytokines and their related genes (total 138 genes, see Table S3 for details; Figure 6A); the second cluster had all transcription factors and their related genes (total 178 genes, see Table S4 for details; Figure 6B). These two clusters revealed that most genes had high expression but low H3K27me3 levels, conversely, a small number of genes had very low expression and their H3K27me3 modifications were high. The results demonstrated that H3K27me3 was associated with inactive genes acting on the promoter regions. Notably, 15.2% cytokine genes and 6.2% TF genes were not negatively regulated by their H3K27me3 modifications (Table 1). For example, high amounts of *CD40*, *NLRP3* and *TFEC* mRNA showed high levels of H3K27me3 modifications at their promoters, while *IL17RC* and *MYT1L* had low mRNA expressions and low H3K27me3 modifications levels. Therefore, H3K27me3 regulates genes expressions but could not permanently repress their expressions [25].

**Table 1:** The list of co-expressed and co-suppressed genes with H3K27me3 modification in the analysis of cytokines and transcription factors genes.

Modification Type	Gene Category	
	Cytokine Genes	TF* Genes
Co-expressed Genes	*C3, CD163, CD40, CD96, IL16, NLRP3, CRTAM, CD300LB, CD8B, IL13RA1*	*MED31, CREBZF, E2F6, ROCK2, GTF2F2, TFEC, TCEAL8*
Co-suppressed Genes	*CD200, IFNAR, IL34, PRG3, CD5L, C16orf5, CD80, DAXX, IL17RC, SCAMP5, CD97*	*TCF4, MYT1L, PKNOX1, IRF3*

* TF: Transcription factors.

### H3K27me3 Modifications Effects on the Parity-specific Differentially Expressed Genes

**Figure 7:**
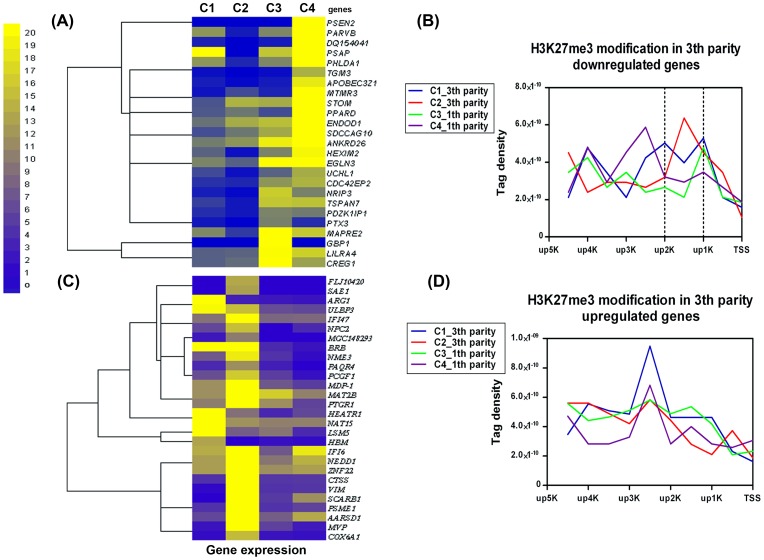
The genes expressions and H3K27me3 modification of DGE differential genes between the two parity cows. A total of 53 differentially expressed genes between the two parity cows were tested (FDR ≤0.01 and | log2Ratio |≥1), in which 28 genes were up-regulated and other 25 genes were down-regulated in the third parity cows. (A) The heatmap of 25 down-expressed genes for the four individuals. Normalized intensity values of genes (rows) were ordered using Centroid Spearman Rank Correlation and hierarchical clustering in Cluster3.0 software. The dendrogram showed the similarity (distance) of mRNA expression levels and was divided into sub-trees as distinguished from different colors. Arrays (columns) were grouped by four different individuals. Yellow and blue colors reflect the high and low expression intensities, respectively. (B) Modifications of H3K27me3 in 25 down-regulated genes for the four individuals. Profiles of the H3K27me3 covered the region of up5K to TSS of the genes were shown. The tag density (number of tags per base pair) was calculated in 500 bp windows in upstream 5 K regions to TSS. (C) The heatmap of 28 up-expressed genes for the four individuals. (D) Modifications of H3K27me3 in 28 up-expressed genes for the four samples.

In order to reveal whether the bovine parity-related genes correlate with H3K27me3 modifications, differentially expressed genes were screened and analyzed between the first and the third parity cows. A total of 53 differentially expressed genes were found (FDR ≤0.01 and | log2Ratio |≥1), in which 25 genes were high expressed in the first parity and low expressed in the third parity (Figure 7A), and the other 28 genes were conversely (Figure 7C). To explore the relationship between H3K27me3 modification and bovine parity-related genes, we calculated H3K27me3 levels in the regions of up5K to TSS of the parity-specific differentially expressed genes. As shown in Figure 7B, the H3K27me3 modification levels of the third-parity-specific down regulated genes were significantly higher in the third parity cows than of the first parity cows, and the remarkable modification region was only located in the up2K-up1K region relative to TSS (*P*<0.05, *t-*test) (Figure 7B). However, the up-regulated genes in the third parity cows did not showed any relationship with H3K27me3 level (Figure 7D).

To explore the function significance of DGE differentially expressed genes regulated by H3K27me3 modification in the third parity cows, we further conducted GO analyses in DAVID database for the 53 differentially expressed genes. The result indicated that *HEXIM2*, *PSEN2* and *PTX3*, *PSAP* genes of down-regulated in the 3rd parity cows were involved in the GO term of transcription regulator, immune response and reproductive process, respectively (Figure S6). Moreover, the pathway analysis for the 25 down-regulated genes was also performed. The result showed that down-regulated genes mainly participated in cancer- and disease-related pathways (Figure S7).

### Real-time PCR Validation

**Figure 8:**
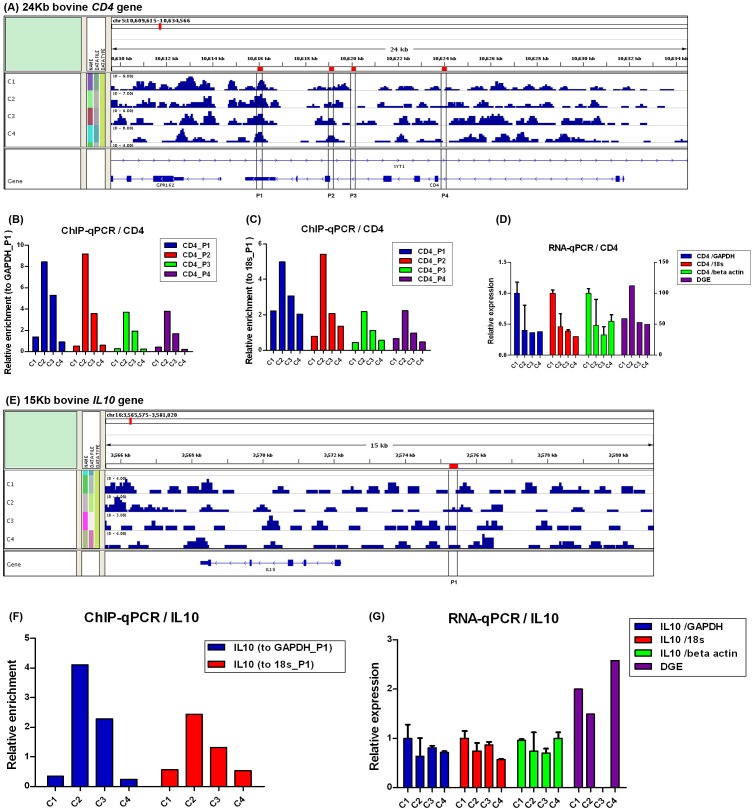
H3K27 trimethylations of *CD4* and *IL10* overlaying different regions and genes expressions in bovine lymphocytes. (A–D) The H3K27me3 enrichment and expression for bovine *CD4* gene were showed in four individuals (C1, C2, C3 and C4). (A) The histone modification profile was shown in custom track in IGV2.0 (Integrative Genomics Viewer). Regions enriched for H3K27me3 in bovine lymphocytes were shown (blue bar). Four sites of *CD4* validated by real-time PCR were indicated (line boxes). The position of the gene was presented on the bottom of the panel, where blue boxes represent exons and the arrow means the transcriptional direction of the gene. (B–C) Real-time PCR results showing enrichment of indicated four sites of *CD4* in H3K27me3 ChIP-seq results carried out in the four cows. The negative controls were *P1* site in the promoter of *GAPDH* (Figure 8B) and *P1* site in the promoter of *18s rRNA* (Figure 8C), respectively. (D) Real-time RT-PCR were performed on the samples of the four cows for validation of *CD4* expression (*GAPDH*, *18s rRNA* and *beta actin* were housekeeping genes). The individual whose Cp value corrected was the minimum was selected as a control sample to calculate relative expression of *CD4* for all four cows. Error bars indicate standard deviation of three technical replicates. DGE indicates the results of digital gene expression of *CD4* (right Y axis for TPM). (E–G) The H3K27me3 enrichment and expression for bovine *IL10* gene were showed in the four individuals. The details are similar to *CD4* gene. A zero-height bar indicates this gene was not expressed in individual C3.

To assess the accuracy of the ChIP-seq mapping result, bovine *CD4* and *IL10* cytokine genes were used to confirm their H3K27me3 enrichment profile using ChIP - quantitative PCR (ChIP-qPCR) approach [34]. We arbitrarily chose five enriched regions from the H3K27me3 maps for the two genes (4 sites on the gene body region of *CD4* and 1 site on the promoter region of *IL10*). Relative enrichment was quantified for each site with real-time PCR reactions using 0.5 ng ChIP DNA or 0.5 ng input DNA for each sample and normalized by the negative controls [34]. For the four sites on *CD4* gene (Figure 8A), the relative enrichments was mostly consistent with the profiles observed in ChIP-seq (Figure 8B and 8C), so did for *IL10* gene (Figure 8E and 8F).

In order to confirm the results of DGE and explore the relationship between H3K27me3 enrichment and gene expression, the expression levels of *CD4* and *IL10* genes were also detected with reverse transcription - quantitative PCR (RT-qPCR) and standardized with three housekeeping genes. The results showed that the expressions of *CD4* and *IL10* were mostly consistent with the data in the DGE (Figure 8D and 8G, except *CD4* expression level in C2 individual in DGE) and were negatively related with the level of H3K27me3. Consequently, the ChIP-seq and DGE data were reliably and efficiently used for the analysis.

## Discussion

Genome-wide measurements for studying the interactions between histone and DNA as well as their regulation on transcriptional activity were increased rapidly by high-throughput DNA sequencing techniques [35], [36]. In the present study, we first described the genome-wide H3K27me3 profiling of peripheral blood lymphocytes in dairy cows. The results indicated that H3K27me3 modification were associated with gene silencing in bovine lymphocytes. Five H3K27me3 modified regions were identified, which included enrichments in the promoter, at the transcriptional start site, at the transcriptional end site, across the gene body region and 3’ region. All regions present in the four Holstein cows depicting that H3K27me3 were enriched mainly in upstream and downstream regulation regions of genes. It was notable that differences of H3K27me3 were observed between the first and the third parity cows. The results provided new insights into gene expression regulated by H3K27me3 modification in bovine peripheral blood lymphocytes.

Lymphocytes are a key part of the immune system and involved in innate and adaptive immunity. The bovine genome-wide patterns of H3K27me3 in peripheral blood lymphocytes are in agreement with previous findings of CD4+ T cells in human [20], and also provided novel information on lymphocytes epigenetic regulation. As previous studies reported, higher H3K27me3 signals in bovine lymphocytes were detected at silent promoters but decreased levels were present at active promoters. In particular, the upstream2Kb region relative to the TSS showed significant correlation with gene silencing, while the upstream region from 2 Kb to 10 Kb relative to TSS of the silent genes as well as the highly, moderately and lowly expressed genes did not present this feature (Figure S5A, S5B, S5C and S5D). The possible reason was that the region of up2K-up10K of the genes might be overlapped with intergenic regions that did not show much modification changes of H3K27me3 [20]. The results indicated that the H3K27me3 modification on 2 Kb upstream of the TSS have a significant regulatory effect on genes suppression in bovine lymphocytes.

Somatic cell count (SCC) is an indicator of milk quality and udder health in dairy herds. General agreement relies on the values of SCCs that less than 100,000 cells/ml for healthy cows, when SCC greater than 300,000/ml is indicated a problem with subclinical or clinical mastitis and inferior milk quality [6]. It was found that SCC increases with increased parity that could be due to increased risk of infectious pathogens entering the udder [37], [38], [39]. Considering the SCC of the third-parity cows (SCC>400,000 cells/ml) significantly higher than the first-parity cows (SCC <150,000 cells/ml) [40], we compared the differences of H3K27me3 modification and gene expression levels between the third-parity cows and the first-parity cows. As expected, the 25 decreased expression genes in the third parity cows were suppressed by the H3K27me3 modification, which were mainly located in the up2K-up1K region relative to TSS of the genes. Among the genes, *PSEN2* (presenilin-2) is extremely correlated with Alzheimer’s disease (AD) risk. Presenilin has been expressed all through the mammal development process and the loss of its function causes neuron apoptosis and impairs long term memory and cognitive deficits in AD patients. For *PSEN1 and* 2 double knock-out mice, loss of presenilins function leads to up-regulation of inflammatory markers in the cerebral cortex [41]. Thus the potential function of silenced *PSEN2* on increased SCC companion with the third-parity cows is worth further study. Unexpected, the down expressed genes involved in immune response in the first parity (but up expressed in the third parity, such as *IFI6* and *IFI47*) cannot be found the regulation effect of H3K27me3 (Figure 7C and 7D), which might be regulated by other epigenetic regulatory machinery such as H3K4me3.

The GO analysis of the differentially expressed genes between the two parities demonstrated that unique third-parity enrichment genes mainly involve in protein-DNA complex and some important immune processes. In the above analysis with cytokines and transcription factors cluster, several genes appeared as parity specific. We assumed that third-parity cows may be infected by subclinical mastitis by the following mechanisms: due to low level of H3K27me3 modification of *IL10*, more IL10 released by cytotoxic T-cells could inhibit the actions of NK cells during the immune response to bacterial infection [42]. These results suggested H3K27me3 levels of vital genes related to immune response could serve as a prognostic factor for udder health of dairy cattle [43].

We applied direct sequencing of ChIP DNA using Illumina Hiseq2000, which is an efficient method for mapping genome-wide distributions of histone modifications and chromatin protein target sites. The technique is particularly well suited for analyzing protein locations and chromatin modification in large genomes, and allows researchers to survey more of the genome in less time. Indeed, we used these techniques to provide the most comprehensive genome-wide data for H3K27me3 marks in bovine lymphocytes. However, in the study, hundreds of peaks in bovine lymphocytes were found, which is far less than that in the human genome [44] and also in the maize genome [45] (see Figure S4). The reason may be associated with species, breeds or samples. As the H3K27me3 modification is highly complex, therefore, further studies are required to determine its function in different kinds of cells and tissues using larger sample sizes.

To the best of our knowledge, we have generated the first high resolution map of histone methylation for bovine lymphocytes. The results not only provides an initial blueprint of bovine H3K27me3 as an epigenetic mark that mediates gene silencing, but also offers a novel view of the complexity of regulating gene expression of lymphocytes. The down-expressed genes in the third parity cows regulated by H3K27me3 modification suggest that the regions of the up2Kb to up1Kb of the TSS are a potent target of epigenetic regulation. Further studies are warranted to better understand the regulation mechanism of the histone methyaltion on SCC increases or milk losses in the latter parity of dairy cattle.

## Materials and Methods

### Cows and Isolation of Lymphocytes

Four Holstein cows (C1, C2, C3 and C4) were randomly selected from a dairy herd in Beijing, China. They were fed on the same lactation diet according to energy recommendations for lactating Chinese Holstein cows and were handled in accordance with the guidelines of the Animal Care and User Committee. Blood samples were collected from the jugular vein for each animal. Performance testing data were provided by the official Dairy Data Center of China (Beijing, China), including daily milk yields, fat percentage, protein percentage and somatic cell counts (SCC) were obtained (Figure1B). In addition, peripheral blood lymphocytes were prepared by Lymphocytes Separation Medium (TBDsciences, Tianjin, China, PN.LTS1086) according to the manufacturer’s instructions, and the purity was 90%–95%.

### ChIP and ChIP-Seq

Lymphocytes from peripheral blood were digested with MNase (TAKARA, D2910) to generate mainly mononucleosomes with a minor fraction of dinucleosomes for histone modification mapping. Chromatin from 5×10^7^ cells were used for ChIP experiment and antibodies against histone H3K27me3 (millipore, Cat. #17–622) were used, that yielded approximately 30 ng of DNA. The enrichment efficiency of ChIP was detected using a standard Real-time Quantitative PCR approach with 1 ng ChIP DNA and non-enriched input DNA (control samples) as template. The ChIP DNA fragments were blunt-ended and ligated to the Solexa adaptors (Paired-End DNA Sample Prep kit, Illumina, Cat. #PE-102–1001), the ChIP DNA was then amplified using the adaptor primers for 15 cycles and the fragments around 100–300 bp (mononucleosome + adaptors) were isolated from agarose gel. The purified DNA was used directly for cluster generation and sequencing analysis using the Illumina Hiseq2000 following manufacturer protocols (BGI, China).

### Solexa Sequencing Data Analysis of ChIP-seq

Sequence reads of 49 bp length of ChIP-seq were obtained using the Solexa Analysis Pipeline that were mapped to the bovine reference genome (Btau4.0) by SOAP 2.21 software and only uniquely matching reads were retained after filtering dirty reads. Unique reads numbers for each library were listed in Figure 2(A).

The output of the Solexa Analysis Pipeline was converted to browser-extensible data (BED) files for viewing the data in the UCSC genome browser. Data for H3K27me3 modifications were presented in both BED and graph format. BED file indicates the genomic coordinates of each tag. To make the graph file, we mapped tags into non-overlapping 50 bp windows of reference genome. The location of a tag on positive or negative strand was determined and represented with the beginning of the tag relative to reference genome. Based on these locations, the tags overlapping each 50 bp summary window were counted.

For ChIP-seq experiments, peak areas represent in vivo locations where proteins of interest (e.g. modified histones or transcription factors) were associated with DNA. We identified such peaks using the “model-based analysis of ChIP-seq data” MACS1.4.0 software, and its function is to isolate ChIP-enriched regions from non-enriched regions based on a dynamic Poisson distribution model. Detailed algorithms and models were described by Zhang et al [46]. We set up a *bandwidth* of 200 bp, *mfold* of 30, *p-value* of 1.00e-05 under a FDR cutoff of 1% to call peaks representing enriched H3K27me3 marks. The output result includes one EXCEL file containing the *genome coordinates*, *summit*, *p-value*, and *fold_enrichment* of each peak. Furthermore, for each gene, as long as there is 1 bp overlap between regions of a peak and a particular gene (includes regions of up20K, exon, intron and down20K) we consider that this peak is associated with this gene.

### Digital Gene Expression and Analysis

Total RNA from primary lymphocytes was extracted with mirVana miRNA Kit (Ambion, PN.AM1560) according to the manufacturer’s instructions. Approximately 6 µg of total RNA was transcribed into double-stranded cDNA through Reverse Transcription Kit (Applied Biosystems, PN.4368814). Sample preparation for digital gene expression profiles was achieved through the Gene Expression Sample Prep Kit (Illumina, Part #1004241) with details as follows. Restriction enzyme *NlaIII* was used to cut the CATG sites of cDNA strand, and Illumina adaptor A was ligated. *Mmel* was used to digest at 17 bp downstream of the CATG site and Illumina adaptor B was ligated at 3' end. Primer GX1 and Primer GX2 were added for PCR for 15 cycles. Then, 95 bp fragments were regained through 6% TBE PAGE (see Figure S2). The DNA was purified followed by sequencing with Illumina Hiseq2000 Analyzer. Consequently, each tunnel generated millions of raw reads with sequencing length of 35 bp, but tags that include CATG site were of 21 bp length.

To determine the position of tags sequences, a more stringent mapping method (SOAP 2.21 software) were adopted [47], [48] to map all tags to the bovine reference genome. Raw sequences were transformed into clean tags after certain steps of data-processing. All clean tags were mapped to the reference sequences and only tags with perfect matching or 1 bp mismatch were considered.

The expression level of one gene was represented by the total number of tags that uniquely aligned to this gene. In order to compare multiple samples differences for one gene, the number of unambiguous and clean tags for each gene was calculated and then normalized to TPM (number of transcript copies in per million clean tags), equaling to copy number of clean tags for this gene divided by total number of clean tags and multiplied by one million [49], [50]. Sequencing quality evaluation is shown in Figure S3.

### Combination Analysis of ChIP-seq with DGE Data

To study the correlation of H3K27me3 modifications with gene expressions, transcriptional levels of genes in bovine lymphocytes were obtained by DGE analysis. These genes (∼7000 genes) were then broken up into 10 sets of 700 genes by ranking these genes expression levels and assigning them to sets of 700 genes. Four out of the ten sets shown in Figures 4 and Figure 5 correspond to highly expressed, two degrees of intermediately expressed (medium and low) and silent genes. Tags detected were aligned in each gene set across transcription start sites (TSS) or gene bodies. For H3K27me3 modification near TSS, the tag density (number of tags per base pair) was calculated in 50 bp windows relative to the TSS. To calculate the H3K27me3 profiles across the gene bodies, the tag numbers detected in every 5% of the gene-body region and every 1 kb outside of the gene-body region were summed and normalized in the four expressed sets.

### Quantitative Real-time PCR

ChIP-q-PCR reactions with SYBR green dye were carried out using Roche LightCycler 480 qPCR machine to confirm the enrichment of selective regions. PCR primer pairs were designed using Primer3 (http://fokker.wi.mit.edu/primer3/input.htm) and confirmed by Oligo 6.0. Primer sequences are given in Table S5. A standard curve was performed using genomic DNA prepared for each primer pair to determinate primer pair efficiency [51], and three serial tenfold dilutions were used ranging from 0.5 ng to 50 ng. Each 20 µL reaction contained 7 µL of nuclease-free water (Gibco), 10 µL of LightCycler 480 SYBR Green I Master mix (Roche, Cat. #04 707 516001), 1 µL of each primer (10 pmol/ µL) and 1 µL of DNA. The ChIP-qPCR reactions were done in duplicates for each site. To determine the relative fold enrichments, the 2^−△△Cp^ method was used by comparing enrichment values for a given primer pair (totally five pairs in *CD4* and *IL10*) to a negative primer pair (*GAPDH_P1* or *18s rRNA_P1*) between experimental (ChIP DNA) and reference (input DNA) samples. The *P1* sites in promoters of *GAPDH* and *18s rRNA* were selected as negative controls that has little H3K27me3 modification and no difference among the experimental samples and control samples (Figure S8) [34].

For RT-qPCR of gene expression, 3 µg of total RNA were reverse transcribed into cDNA with High-Capacity cDNA Reverse Transcription Kit (Applied Biosystems, PN.4368814) according to the recommended procedure. Triplicates were performed for RT-qPCR reactions. qPCR reaction was run using the program as follows: pre-incubation (95°C for 10 min), 45 cycles of amplification (95°C for 10 s, 60°C for 10 s, and 72°C for 10s), melting curves using a heat ramp and cool down. Crossing point values (Cp values) were obtained using the second derivative maximal analysis tool included in the Roche LightCycler480 software. The mRNA expression of *CD4* and *IL10* was normalized against three housekeeping genes (*GAPDH*, *18s rRNA* and *beta actin*) cDNA in the corresponding samples.

## Supporting Information

Figure S1 **Scaling analysis of H3K27me3 peaks.** The tag density on per peak of the sample was plotted. By increasing the fraction of tags selected for peak identification, a degree of saturation was estimated based on number of tags per base pair on the peak.
(DOCX)

Figure S2 **Principle and procedure of Tag preparation for DGE pipeline** [**49**] **.** Beads of Oligo(dT) are used to enrich mRNA from the total RNA, and then are synthesized to the first and second-strand cDNA by use of Oligo(dT) as primer. Subsequently restriction enzyme *Nla?* recognizes and cut off the CATG sites of cDNA strand, and the Illumina adaptor A is ligated to the sticky 5' end. In the same, *Mme?* which is a type of Endonuclease cut at 17 bp downstream of the CATG site, and the Illumina adaptor B is ligated to the 3’ ends of tags, consequently, a tag library that tags with different adaptors of both ends is acquired. After 15 cycles of linear PCR amplification, 95 bp fragments are purified by 6% TBE PAGE Gel electrophoresis. The purified fragments were used directly for cluster generation and sequencing analysis using the Illumina Hiseq2000 following manufacturer protocols (BGI, China).
(DOCX)

Figure S3 **Distribution of Tag Expression in DGE data.** The upper panel is the total tag number of sample C1, C2, C3 and C4. For example, “Tags Containing N (209886, 3.75%)” means the number of tags containing N is 209886 and 3.75% of the total tags. The under panel is the distinct tags number of sample C1, C2, C3 and C4. For example, “Tags Containing N (92427, 9.61%)” means the number of the distinct tags containing N is 92427 and 9.61% of the total distinct tags; “Only adaptors” means the reads contain only the adaptors sequence; “Copy Number <2” is the tags whose copy number is less than 2; “Clean tags” is the tags used to analysis after filtering the dirty tags. Raw sequences have 3' adaptor fragments as well as a few low-quality sequences and several types of impurities. Raw sequences are transformed into Clean Tags after certain steps of data-processing.
(DOCX)

Figure S4 **H3K27me3 Peaks information.** (A) to (C) Peaks Numbers, average lengths, and total lengths of epigenetically modified regions detected by MACS1.4.0 software, respectively.
(DOCX)

Figure S5 **H3K27me3 modification in four different expressed sets.** Profiles of the H3K27me3 covered the region of upstream 10 K to TSS for highly active (A), two kinds of intermediately active (medium (B) and low (C)) and silent gene (D) sets were shown. Each gene set included common genes shared by four individuals (C1, C2, C3 and C4), which were screened from 700 genes in Figure 4 or Figure 5. Here, the tag density (number of tags per base pair) was calculated in 50 bp windows in upstream 10 K regions to TSS (see Experimental Procedures).
(DOCX)

Figure S6 **Annotation of DGE differential genes for two parities by WEGO.** Gene Ontology Annotation Plotting. The BGI WEGO (Web Gene Ontology Annotation Plotting) was used to functionally categorize parity-specific differentially expressed genes. Of 53 differentially expressed genes, 43 genes with GO annotation that belong to two parities were grouped by cell component, molecular function and biological process based on the bovine GO annotation information (http://www.geneontology.org/GO.downloads.annotations.shtml). Gene numbers and percentages (on log scale) are listed for each category.
(DOCX)

Figure S7 **The pathway analysis of down-regulated genes in the third parity.** KEGG pathway analysis in DAVID for the 25 down-regulated genes was completed. Y axis indicated pathway terms of involving in these down-regulated genes.
(DOCX)

Figure S8 **H3K27me3 profiles of** ***GAPDH*** **and** ***18s rRNA*** **.** (A) The H3K27me3 enrichment of ChIP-seq for bovine *GAPDH* gene was shown in four individuals (C1, C2, C3 and C4). The *P1* site of negative control was indicated (line boxes), which locates in upstream 500 bp of transcription start site (TSS: 110663704) of the gene. (B) The H3K27me3 enrichment of ChIP-seq for bovine *18s rRNA* gene was shown. The *P1* site of negative control was indicated (line boxes), which locates in upstream 300 bp of transcription start site (TSS: 18278744). (C-D) Real-time PCR results showing enrichment of indicated two sites of *GAPDH* (C) and *18s rRNA* (D) in H3K27me3 ChIP-seq results carried out in the four cows. Y axis represents the crossing point values of real-time qPCR in the P1 sites of *GAPDH* and *18s rRNA*. The results showed no significant difference between experiments (ChIP DNA samples) and references (input DNA samples).
(DOCX)

Table S1 Supporting file for Figure4-H3K27me3 modification relative to TSS.
(XLSX)

Table S2 Supporting file for Figure5-H3K27me3 modification across gene body.
(XLSX)

Table S3 Supporting file for Figure6A-Cytokines genes cluster.
(XLSX)

Table S4 Supporting file for Figure6B-Transcription factors genes cluster.
(XLSX)

Table S5 Primers sequences for ChIP-qPCR and RT- qPCR analyses.
(DOCX)

## References

[1] Hare E, Norman HD, Wright JR (2006). Survival rates and productive herd life of dairy cattle in the United States.. J Dairy Sci.

[2] Caraviello DZ, Weigel KA, Shook GE, Ruegg PL (2005). Assessment of the impact of somatic cell count on functional longevity in Holstein and Jersey cattle using survival analysis methodology.. J Dairy Sci.

[3] Hultgren J, Svensson C (2009). Lifetime risk and cost of clinical mastitis in dairy cows in relation to heifer rearing conditions in southwest Sweden.. J Dairy Sci.

[4] Hultgren J, Svensson C (2009). Heifer rearing conditions affect length of productive life in Swedish dairy cows.. Prev Vet Med.

[5] ten Napel J, de Haas Y, de Jong G, Lam TJ, Ouweltjes W (2009). Characterization of distributions of somatic cell counts.. J Dairy Sci.

[6] Rupp R, Beaudeau F, Boichard D (2000). Relationship between milk somatic-cell counts in the first lactation and clinical mastitis occurrence in the second lactation of French Holstein cows.. Prev Vet Med.

[7] Rivas AL, Quimby FW, Coksaygan O, Olmstead L, Lein DH (2000). Longitudinal evaluation of CD4+ and CD8+ peripheral blood and mammary gland lymphocytes in cows experimentally inoculated with Staphylococcus aureus.. Can J Vet Res.

[8] Ohtsuka H, Terasawa S, Watanabe C, Kohiruimaki M, Mukai M (2010). Effect of parity on lymphocytes in peripheral blood and colostrum of healthy Holstein dairy cows.. Can J Vet Res.

[9] Kioussis D, Georgopoulos K (2007). Epigenetic flexibility underlying lineage choices in the adaptive immune system.. Science.

[10] Alcobia I, Quina AS, Neves H, Clode N, Parreira L (2003). The spatial organization of centromeric heterochromatin during normal human lymphopoiesis: evidence for ontogenically determined spatial patterns.. Experimental Cell Research.

[11] Kuchen S, Resch W, Yamane A, Kuo N, Li Z (2010). Regulation of microRNA expression and abundance during lymphopoiesis.. Immunity.

[12] Crispi KT, Mallard BA, Heriazon A, Hine B, Hussey B (2010). Genetic and Epigenetic Effects on Bovine Immune Responses and their Implications to Dairy Health.. Proc 9th World Congress Genetics Applied to Livestock Prod Aug 1–6, Leipzig (Germany) 1–521.

[13] Woo HD, Kim J (2012). Global DNA Hypomethylation in Peripheral Blood Leukocytes as a Biomarker for Cancer Risk: A Meta- Analysis.. PLoS ONE.

[14] Hamatani T, Falco G, Akutsu H, Stagg CA, Sharov AA (2004). Age-associated alteration of gene expression patterns in mouse oocytes.. Human Molecular Genetics.

[15] Singh K, Erdman RA, Swanson KM, Molenaar AJ, Maqbool NJ (2010). Epigenetic regulation of milk production in dairy cows.. J Mammary Gland Biol Neoplasia.

[16] Berger SL (2002). Histone modifications in transcriptional regulation.. Current Opinion in Genetics & Development.

[17] Greer EL, Maures TJ, Ucar D, Hauswirth AG, Mancini E (2011). Transgenerational epigenetic inheritance of longevity in Caenorhabditis elegans.. Nature.

[18] Young MD, Willson TA, Wakefield MJ, Trounson E, Hilton DJ (2011). ChIP-seq analysis reveals distinct H3K27me3 profiles that correlate with transcriptional activity.. Nucleic Acids Res.

[19] Derks S, Bosch LJ, Niessen HE, Moerkerk PT, van den Bosch SM (2009). Promoter CpG island hypermethylation- and H3K9me3 and H3K27me3-mediated epigenetic silencing targets the deleted in colon cancer (DCC) gene in colorectal carcinogenesis without affecting neighboring genes on chromosomal region 18q21.. Carcinogenesis.

[20] Barski A, Cuddapah S, Cui K, Roh TY, Schones DE (2007). High-resolution profiling of histone methylations in the human genome.. Cell.

[21] Hansen KH, Bracken AP, Pasini D, Dietrich N, Gehani SS (2008). A model for transmission of the H3K27me3 epigenetic mark.. Nature Cell Biology.

[22] Breton A, LE Bourhis D, Audouard C, Vignon X, Lelievre JM (2010). Nuclear Profiles of H3 Histones Trimethylated on Lys27 in Bovine (Bos taurus) Embryos Obtained after In Vitro Fertilization or Somatic Cell Nuclear Transfer.. Journal of Reproduction and Development.

[23] Yu HQ, Wu X, Li Y, Xue LA, Wang LL (2011). Multiple histone site epigenetic modifications in nuclear transfer and in vitro fertilized bovine embryos.. Zygote.

[24] Ross PJ, Ragina NP, Rodriguez RM, Iager AE, Siripattarapravat K (2008). Polycomb gene expression and histone H3 lysine 27 trimethylation changes during bovine preimplantation development.. Reproduction (Cambridge, England).

[25] Wei G, Wei L, Zhu JF, Zang CZ, Hu-Li J (2009). Global Mapping of H3K4me3 and H3K27me3 Reveals Specificity and Plasticity in Lineage Fate Determination of Differentiating CD4(+) T Cells.. Immunity.

[26] Fouse SD, Shen Y, Pellegrini M, Cole S, Meissner A (2008). Promoter CpG methylation contributes to ES cell gene regulation in parallel with Oct4/Nanog, PcG complex, and histone H3 K4/K27 trimethylation.. Cell stem cell.

[27] Pan GJ, Tian SL, Nie J, Yang CH, Ruotti V (2007). Whole-genome analysis of histone H3 lysine 4 and lysine 27 methylation in human embryonic stem cells.. Cell stem cell.

[28] Nix DA, Courdy SJ, Boucher KM (2008). Empirical methods for controlling false positives and estimating confidence in ChIP-Seq peaks.. Bmc Bioinformatics.

[29] Szalkowski AM, Schmid CD (2010). Rapid innovation in ChIP-seq peak-calling algorithms is outdistancing benchmarking efforts.. Briefings in Bioinformatics.

[30] Boyer LA, Plath K, Zeitlinger J, Brambrink T, Medeiros LA (2006). Polycomb complexes repress developmental regulators in murine embryonic stem cells.. Nature.

[31] Roh TY, Cuddapah S, Cui KR, Zhao KJ (2006). The genomic landscape of histone modifications in human T cells.. Proceedings of the National Academy of Sciences of the United States of America.

[32] Kouzarides T (2007). Chromatin modifications and their function.. Cell.

[33] Lee CG, Sahoo A, Im SH (2009). Epigenetic regulation of cytokine gene expression in T lymphocytes.. Yonsei Med J.

[34] Lefrancois P, Euskirchen GM, Auerbach RK, Rozowsky J, Gibson T (2009). Efficient yeast ChIP-Seq using multiplex short-read DNA sequencing.. BMC Genomics.

[35] Kircher M, Kelso J (2010). High-throughput DNA sequencing–concepts and limitations.. Bioessays.

[36] Johnson DS, Mortazavi A, Myers RM, Wold B (2007). Genome-wide mapping of in vivo protein-DNA interactions.. Science.

[37] Walsh S, Buckley F, Berry DP, Rath M, Pierce K (2007). Effects of breed, feeding system, and parity on udder health and milking characteristics.. Journal of Dairy Science.

[38] Koc A (2008). A study of somatic cell counts in the milk of Holstein-Friesian cows managed in Mediterranean climatic conditions.. Turkish Journal of Veterinary & Animal Sciences.

[39] Dürr JW, Cue RI, Monardes HG, Moro-Méndez J, Wade KM (2008). Milk losses associated with somatic cell counts per breed, parity and stage of lactation in Canadian dairy cattle.. Livestock Science.

[40] Xu M, Ping FQ, Chen SY, Lai SJ, Liu YP (2008). Study on the relationships between polymorphisms of CXCR2 gene and milk quality and mastitis of dairy cow.. Hereditas (China).

[41] Beglopoulos V, Sun X, Saura CA, Lemere CA, Kim RD (2004). Reduced beta-amyloid production and increased inflammatory responses in presenilin conditional knock-out mice.. J Biol Chem.

[42] Vivier E, Ugolini S (2009). Regulatory Natural Killer Cells: New Players in the IL-10 Anti-Inflammatory Response.. Cell Host & Microbe.

[43] Wei YK, Xia WY, Zhang ZH, Liu JS, Wang HM (2008). Loss of trimethylation at lysine 27 of histone H3 is a predictor of poor outcome in breast, ovarian, and pancreatic cancers.. Molecular carcinogenesis.

[44] Cui KR, Zang CZ, Roh TY, Schones DE, Childs RW (2009). Chromatin Signatures in Multipotent Human Hematopoietic Stem Cells Indicate the Fate of Bivalent Genes during Differentiation.. Cell stem cell.

[45] Wang XF, Elling AA, Li XY, Li N, Peng ZY (2009). Genome-Wide and Organ-Specific Landscapes of Epigenetic Modifications and Their Relationships to mRNA and Small RNA Transcriptomes in Maize.. Plant Cell.

[46] Zhang Y, Liu T, Meyer CA, Eeckhoute J, Johnson DS (2008). Model-based Analysis of ChIP-Seq (MACS).. Genome Biology.

[47] Li RQ, Li YR, Kristiansen K, Wang J (2008). SOAP: short oligonucleotide alignment program.. Bioinformatics.

[48] Li H, Ruan J, Durbin R (2008). Mapping short DNA sequencing reads and calling variants using mapping quality scores.. Genome Research.

[49] Morrissy AS, Morin RD, Delaney A, Zeng T, McDonald H (2009). Next-generation tag sequencing for cancer gene expression profiling.. Genome Research.

[50] t Hoen PAC, Ariyurek Y, Thygesen HH, Vreugdenhil E, Vossen RHAM (2008). Deep sequencing-based expression analysis shows major advances in robustness, resolution and inter-lab portability over five microarray platforms.. Nucleic Acids Research 36.

[51] Pfaffl MW (2001). A new mathematical model for relative quantification in real-time RT-PCR.. Nucleic Acids Research.

