# Supplementary material for: Genome-Wide Bovine H3K27me3 Modifications and the Regulatory Effects on Genes Expressions in Peripheral Blood Lymphocytes
Source: PLoS One. 2012 Jun 28;7(6):e39094. doi: 10.1371/journal.pone.0039094 (PMC3386284; doi:10.1371/journal.pone.0039094)
Supplement: Figure S1 — Scaling analysis of H3K27me3 peaks. The tag density on per peak of the sample was plotted. By increasing the fraction of tags selected for peak identification, a degree of saturation was estimated based on number of tags per base pair on the peak. (DOCX) [file pone.0039094.s001.docx]

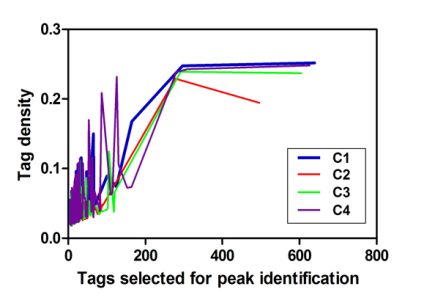


**FigureS1.** **Scaling analysis of H3K27me3 peaks.**

The tag density on per peak of the sample was plotted. By increasing the fraction of tags selected for peak identification, a degree of saturation was estimated based on number of tags per base pair on the peak.
