# Supplementary material for: Genome-Wide Bovine H3K27me3 Modifications and the Regulatory Effects on Genes Expressions in Peripheral Blood Lymphocytes
Source: PLoS One. 2012 Jun 28;7(6):e39094. doi: 10.1371/journal.pone.0039094 (PMC3386284; doi:10.1371/journal.pone.0039094)
Supplement: Figure S2 — Principle and procedure of Tag preparation for DGE pipeline [49] . Beads of Oligo(dT) are used to enrich mRNA from the total RNA, and then are synthesized to the first and second-strand cDNA by use of Oligo(dT) as primer. Subsequently restriction enzyme Nla? recognizes and cut off the CATG sites of cDNA strand, and the Illumina adaptor A is ligated to the sticky 5' end. In the same, Mme? which is a type of Endonuclease cut at 17 bp downstream of the CATG site, and the Illumina adaptor B is ligated to the 3’ ends of tags, consequently, a tag library that tags with different adaptors of both ends is acquired. After 15 cycles of linear PCR amplification, 95 bp fragments are purified by 6% TBE PAGE Gel electrophoresis. The purified fragments were used directly for cluster generation and sequencing analysis using the Illumina Hiseq2000 following manufacturer protocols (BGI, China). (DOCX) [file pone.0039094.s002.docx]

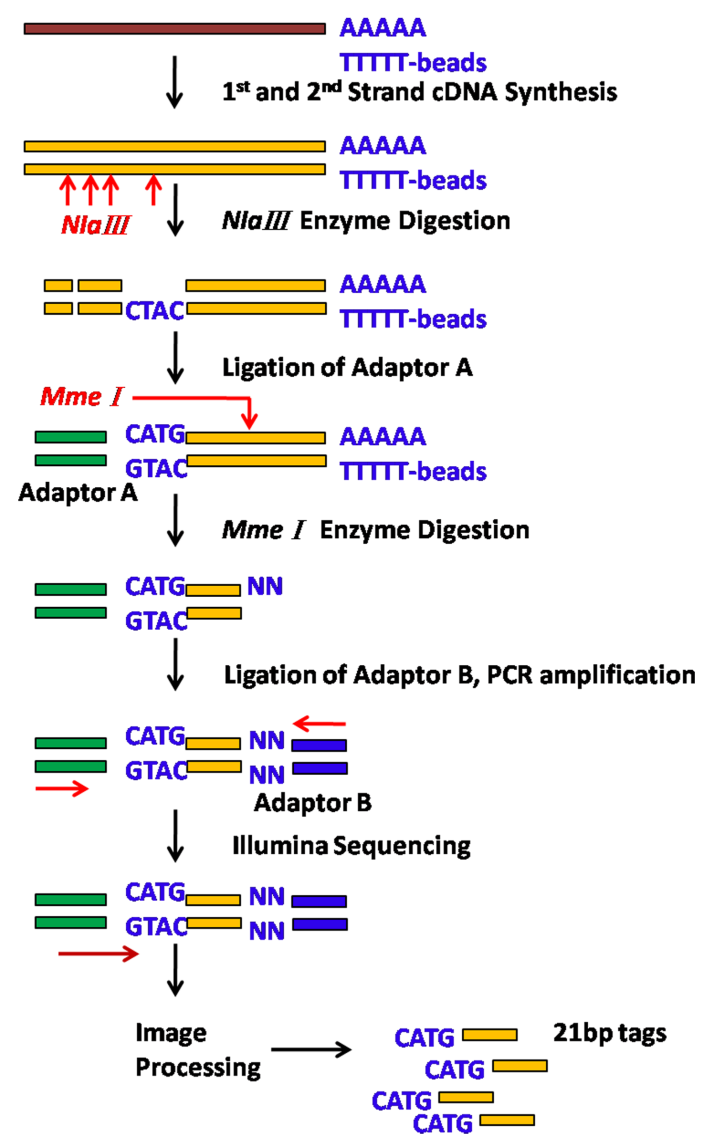


**FigureS2. Principle and procedure of Tag preparation for DGE pipeline [1]**.

Beads of Oligo(dT) are used to enrich mRNA from the total RNA, and then are synthesized to the first and second-strand cDNA by use of Oligo(dT) as primer. Subsequently restriction enzyme *NlaⅢ* recognizes and cut off the CATG sites of cDNA strand, and the Illumina adaptor A is ligated to the sticky 5' end. In the same, *MmeⅠ* which is a type of Endonuclease cut at 17bp downstream of the CATG site, and the Illumina adaptor B is ligated to the 3’ ends of tags, consequently, a tag library that tags with different adaptors of both ends is acquired. After 15 cycles of linear PCR amplification, 95bp fragments are purified by 6% TBE PAGE Gel electrophoresis. The purified fragments were used directly for cluster generation and sequencing analysis using the Illumina Hiseq2000 following manufacturer protocols (BGI, China).

SUPPLEMENTAL REFERENCE

1. Morrissy AS, Morin RD, Delaney A, Zeng T, McDonald H, et al. (2009) Next-generation tag sequencing for cancer gene expression profiling. Genome Research 19(10):1825-35.
