# Supplementary material for: Genome-Wide Bovine H3K27me3 Modifications and the Regulatory Effects on Genes Expressions in Peripheral Blood Lymphocytes
Source: PLoS One. 2012 Jun 28;7(6):e39094. doi: 10.1371/journal.pone.0039094 (PMC3386284; doi:10.1371/journal.pone.0039094)
Supplement: Figure S3 — Distribution of Tag Expression in DGE data. The upper panel is the total tag number of sample C1, C2, C3 and C4. For example, “Tags Containing N (209886, 3.75%)” means the number of tags containing N is 209886 and 3.75% of the total tags. The under panel is the distinct tags number of sample C1, C2, C3 and C4. For example, “Tags Containing N (92427, 9.61%)” means the number of the distinct tags containing N is 92427 and 9.61% of the total distinct tags; “Only adaptors” means the reads contain only the adaptors sequence; “Copy Number <2” is the tags whose copy number is less than 2; “Clean tags” is the tags used to analysis after filtering the dirty tags. Raw sequences have 3' adaptor fragments as well as a few low-quality sequences and several types of impurities. Raw sequences are transformed into Clean Tags after certain steps of data-processing. (DOCX) [file pone.0039094.s003.docx]

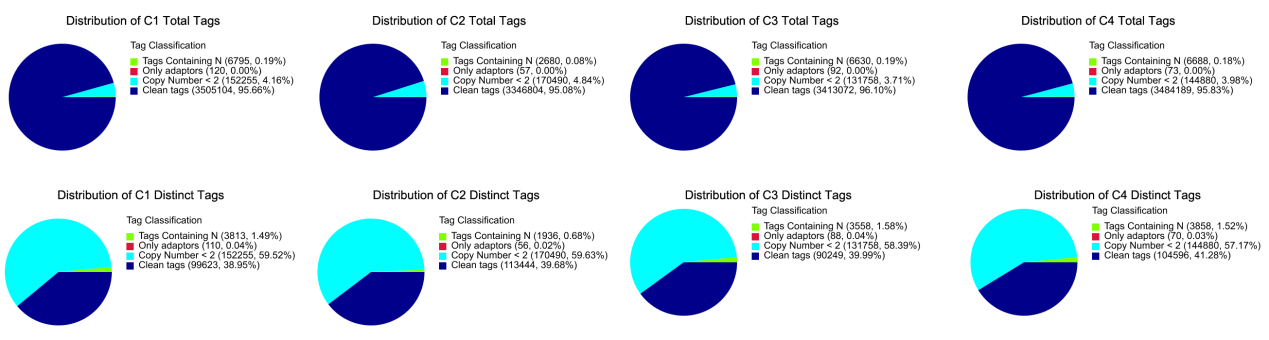


**Figure S3. Distribution of Tag Expression in DGE data.**

The upper panel is the total tag number of sample C1, C2, C3 and C4. For example, “Tags Containing N (209886, 3.75%)” means the number of tags containing N is 209886 and 3.75% of the total tags. The under panel is the distinct tags number of sample C1, C2, C3 and C4. For example, “Tags Containing N (92427, 9.61%)” means the number of the distinct tags containing N is 92427 and 9.61% of the total distinct tags; “Only adaptors” means the reads contain only the adaptors sequence; “Copy Number < 2” is the tags whose copy number is less than 2; “Clean tags” is the tags used to analysis after filtering the dirty tags. Raw sequences have 3' adaptor fragments as well as a few low-quality sequences and several types of impurities. Raw sequences are transformed into Clean Tags after certain steps of data-processing.
