# Supplementary material for: Genome-Wide Bovine H3K27me3 Modifications and the Regulatory Effects on Genes Expressions in Peripheral Blood Lymphocytes
Source: PLoS One. 2012 Jun 28;7(6):e39094. doi: 10.1371/journal.pone.0039094 (PMC3386284; doi:10.1371/journal.pone.0039094)
Supplement: Figure S4 — H3K27me3 Peaks information. (A) to (C) Peaks Numbers, average lengths, and total lengths of epigenetically modified regions detected by MACS1.4.0 software, respectively. (DOCX) [file pone.0039094.s004.docx]

**
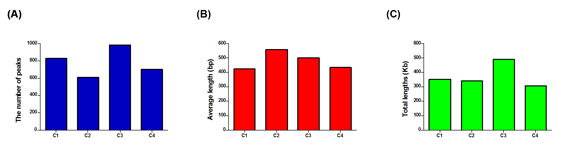
**

**Figure S4. H3K27me3 Peaks information.**

(A) to (C) Peaks Numbers, average lengths, and total lengths of epigenetically modified regions detected by MACS1.4.0 software, respectively.
