# Supplementary material for: Genome-Wide Bovine H3K27me3 Modifications and the Regulatory Effects on Genes Expressions in Peripheral Blood Lymphocytes
Source: PLoS One. 2012 Jun 28;7(6):e39094. doi: 10.1371/journal.pone.0039094 (PMC3386284; doi:10.1371/journal.pone.0039094)
Supplement: Figure S5 — H3K27me3 modification in four different expressed sets. Profiles of the H3K27me3 covered the region of upstream 10 K to TSS for highly active (A), two kinds of intermediately active (medium (B) and low (C)) and silent gene (D) sets were shown. Each gene set included common genes shared by four individuals (C1, C2, C3 and C4), which were screened from 700 genes in Figure 4 or Figure 5. Here, the tag density (number of tags per base pair) was calculated in 50 bp windows in upstream 10 K regions to TSS (see Experimental Procedures). (DOCX) [file pone.0039094.s005.docx]

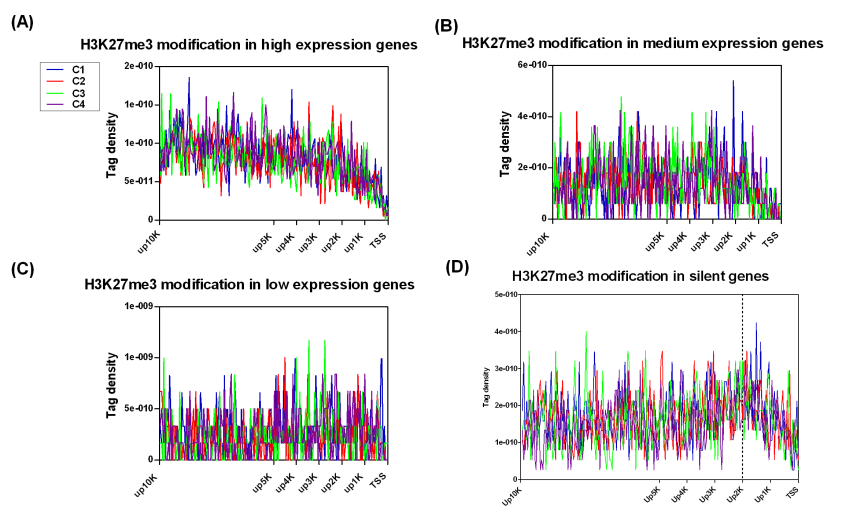


**Figure S5. H3K27me3 modification in four different expressed sets.**

Profiles of the H3K27me3 covered the region of upstream 10K to TSS for highly active (A), two kinds of intermediately active (medium (B) and low (C)) and silent gene (D) sets were shown. Each gene set included common genes shared by four individuals (C1, C2, C3 and C4), which were screened from 700 genes in Figure 4 or Figure 5. Here, the tag density (number of tags per base pair) was calculated in 50 bp windows in upstream 10K regions to TSS (see Experimental Procedures).
