# Supplementary material for: Genome-Wide Bovine H3K27me3 Modifications and the Regulatory Effects on Genes Expressions in Peripheral Blood Lymphocytes
Source: PLoS One. 2012 Jun 28;7(6):e39094. doi: 10.1371/journal.pone.0039094 (PMC3386284; doi:10.1371/journal.pone.0039094)
Supplement: Figure S6 — Annotation of DGE differential genes for two parities by WEGO. Gene Ontology Annotation Plotting. The BGI WEGO (Web Gene Ontology Annotation Plotting) was used to functionally categorize parity-specific differentially expressed genes. Of 53 differentially expressed genes, 43 genes with GO annotation that belong to two parities were grouped by cell component, molecular function and biological process based on the bovine GO annotation information (http://www.geneontology.org/GO.downloads.annotations.shtml). Gene numbers and percentages (on log scale) are listed for each category. (DOCX) [file pone.0039094.s006.docx]

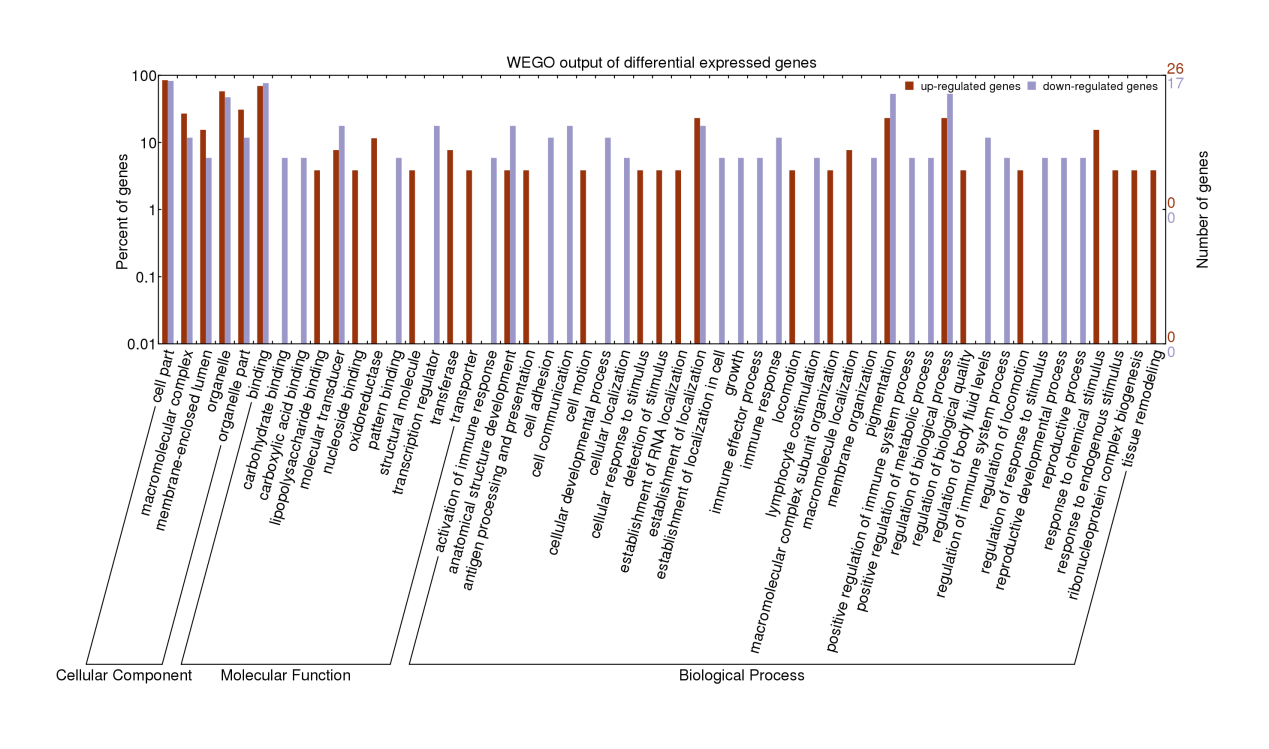


**Figure S6. Annotation of DGE differential genes for two parities by WEGO.**

Gene Ontology Annotation Plotting. The BGI WEGO (Web Gene Ontology Annotation Plotting) was used to functionally categorize parity-specific differentially expressed genes. Of 53 differentially expressed genes, 43 genes with GO annotation that belong to two parities were grouped by cell component, molecular function and biological process based on the bovine GO annotation information (http://www.geneontology.org/GO.downloads.annotations.shtml). Gene numbers and percentages (on log scale) are listed for each category.
