# Supplementary material for: Genome-Wide Bovine H3K27me3 Modifications and the Regulatory Effects on Genes Expressions in Peripheral Blood Lymphocytes
Source: PLoS One. 2012 Jun 28;7(6):e39094. doi: 10.1371/journal.pone.0039094 (PMC3386284; doi:10.1371/journal.pone.0039094)
Supplement: Figure S7 — The pathway analysis of down-regulated genes in the third parity. KEGG pathway analysis in DAVID for the 25 down-regulated genes was completed. Y axis indicated pathway terms of involving in these down-regulated genes. (DOCX) [file pone.0039094.s007.docx]

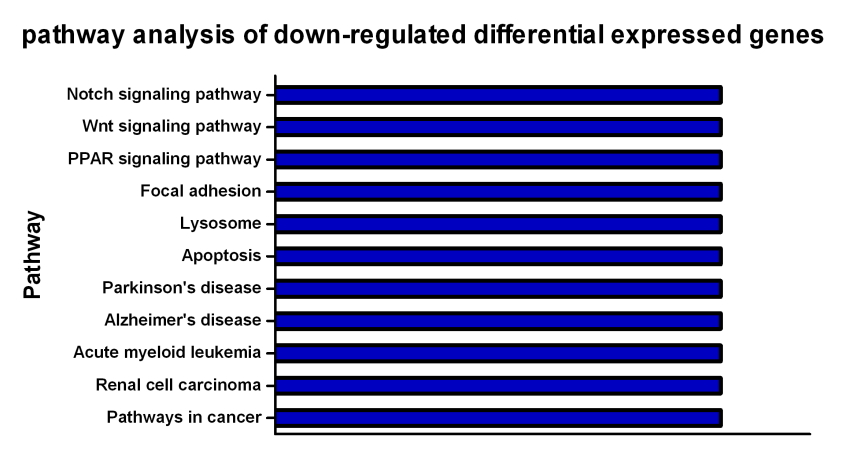


**Figure S7. The pathway analysis of down-regulated genes in the third parity.**

KEGG pathway analysis in DAVID for the 25 down-regulated genes was completed. Y axis indicated pathway terms of involving in these down-regulated genes.
