# Supplementary material for: Genome-Wide Bovine H3K27me3 Modifications and the Regulatory Effects on Genes Expressions in Peripheral Blood Lymphocytes
Source: PLoS One. 2012 Jun 28;7(6):e39094. doi: 10.1371/journal.pone.0039094 (PMC3386284; doi:10.1371/journal.pone.0039094)
Supplement: Figure S8 — H3K27me3 profiles of GAPDH and 18s rRNA . (A) The H3K27me3 enrichment of ChIP-seq for bovine GAPDH gene was shown in four individuals (C1, C2, C3 and C4). The P1 site of negative control was indicated (line boxes), which locates in upstream 500 bp of transcription start site (TSS: 110663704) of the gene. (B) The H3K27me3 enrichment of ChIP-seq for bovine 18s rRNA gene was shown. The P1 site of negative control was indicated (line boxes), which locates in upstream 300 bp of transcription start site (TSS: 18278744). (C-D) Real-time PCR results showing enrichment of indicated two sites of GAPDH (C) and 18s rRNA (D) in H3K27me3 ChIP-seq results carried out in the four cows. Y axis represents the crossing point values of real-time qPCR in the P1 sites of GAPDH and 18s rRNA. The results showed no significant difference between experiments (ChIP DNA samples) and references (input DNA samples). (DOCX) [file pone.0039094.s008.docx]

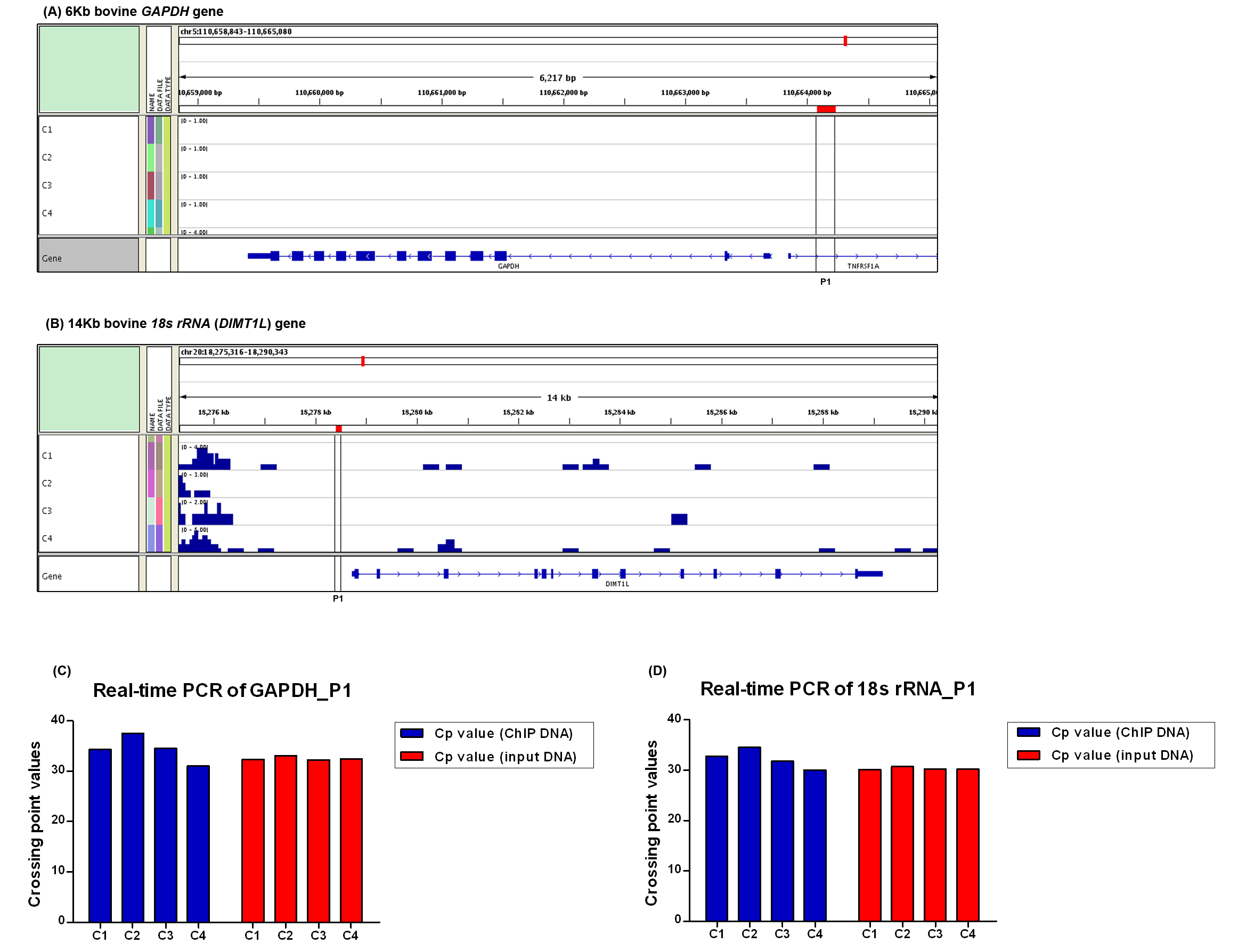


**Figure S8. H3K27me3 profiles of *GAPDH* and *18s rRNA*.**

(A) The H3K27me3 enrichment of ChIP-seq for bovine *GAPDH* gene was shown in four individuals (C1, C2, C3 and C4). The *P1* site of negative control was indicated (line boxes), which locates in upstream 500bp of transcription start site (TSS: 110663704) of the gene..

(B) The H3K27me3 enrichment of ChIP-seq for bovine *18s rRNA* gene was shown. The *P1* site of negative control was indicated (line boxes), which locates in upstream 300bp of transcription start site (TSS: 18278744).

(C-D) Real-time PCR results showing enrichment of indicated two sites of *GAPDH* (C) and *18s rRNA* (D) in H3K27me3 ChIP-seq results carried out in the four cows. Y axis represents the crossing point values of real-time qPCR in the P1 sites of *GAPDH* and *18s rRNA*. The results showed no significant difference between experiments (ChIP DNA samples) and references (input DNA samples).
