# Supplementary material for: Genome-Wide Bovine H3K27me3 Modifications and the Regulatory Effects on Genes Expressions in Peripheral Blood Lymphocytes
Source: PLoS One. 2012 Jun 28;7(6):e39094. doi: 10.1371/journal.pone.0039094 (PMC3386284; doi:10.1371/journal.pone.0039094)
Supplement: Table S5 — Primers sequences for ChIP-qPCR and RT- qPCR analyses. (DOCX) [file pone.0039094.s013.docx]

**Table S1. Primers sequences for ChIP-qPCR and RT- qPCR analyses.**

| qPCR experiment | Primer name | Sequence | Product Size (bp) | Product Type | Location | Annealing Temperature (℃) |
| --- | --- | --- | --- | --- | --- | --- |
| CD4_ChIP | CD4_P1 | F：5' TGG ACT AGC CCA GGT CTC TT 3'  R：5' GCA GGT TTT GTT CCC TGG TA 3' | 249 | DNA | Exon1 | 60° |
| CD4_ChIP | CD4_P2 | F: 5' GAA GCT GTG CTT CCT CCA TT 3'  R: 5' CCA TCT TCA AGT TCA GGG TCA 3' | 223 | DNA | Exon2, Intron2 | 60° |
| CD4_ChIP | CD4_P3 | F: 5' ACT GCA AGG AGA CCC AAC C 3'  R: 5' CCA GCC ATC TCA TCC TCT GT 3' | 193 | DNA | Intron2 | 60° |
| CD4_ChIP | CD4_P4 | F: 5' GGA GAA TCC CAT GGA CAG AA 3'  R: 5' TCT GCC CTG GTC CTA ATC TG 3' | 221 | DNA | Intron5 | 60° |
| IL10_ChIP | IL10_P1 | F: 5' TTC CCA AAA TGT GCA TAC CTC 3'  R: 5' CTT AGC AGC AGC AGC CAA TC 3' | 248 | DNA | Promoter | 60° |
| GAPDH_ChIP | GAPDH_P1 | F: 5' TAC CGA AGA GCC TCG AGA A 3'  R: 5' GCC GCA AGG ATA TAA CAG GA 3' | 155 | DNA | Promoter | 60° |
| 18s rRNA_ChIP | 18s rRNA_P1 | F: 5' ACG TAA CTT AAG CTT CCT CC 3'  R: 5' CGA TTC TAG CAG TTT GGA TT 3' | 125 | DNA | Promoter | 60° |
| CD4_mRNA^*^ | CD4_m | F：5' ACT GAG CCA TCG AGT GGA AT 3'  R：5' CAG TGA CAG GCT CTT GAC GT 3' | 289 | cDNA | - | 60° |
| IL10_mRNA^*^ | IL10_m | F: 5' TGT TGA CCC AGT CTC TGC TG 3'  R: 5' AGC TTC TCC CCC AGT GAG TT 3' | 154 | cDNA | - | 60° |
| GAPDH_mRNA^*^ | GAPDH_m | F: 5’ GGCGTGAACCACGAGAAGTATAA 3’  R: 5’ CCCTCCACGATGCCAAAGT 3’ | 119 | cDNA | - | 60° |
| 18s rRNA_mRNA^*^ | 18s rRNA_m | F: 5’ GTAACCCGTTGAACCCCATT 3’  R: 5’ CCATCCAATCGGTAGTAGCG 3’ | 152 | cDNA | - | 60° |
| beta actin_mRNA^*^ | beta actin_m | F: 5’ AGCAAGCAGGAGTACGATGAGT 3’ R: 5’ ATCCAACCGACTGCTGTCA 3’ | 239 | cDNA | - | 60° |

* Samples were prepared to validate gene expression levels, and cDNA transferred from total RNA were used as templates for real-time PCR.
